# Baicalin Enhanced Oral Bioavailability of Sorafenib in Rats by Inducing Intestine Absorption

**DOI:** 10.3389/fphar.2021.761763

**Published:** 2021-11-08

**Authors:** Jingyao Wei, Ruijuan Liu, Jiali Zhang, Shuaibing Liu, Dan Yan, Xueqian Wen, Xin Tian

**Affiliations:** ^1^ Department of Pharmacy, The First Affiliated Hospital of Zhengzhou University, Zhengzhou, China; ^2^ Henan Key Laboratory of Precision Clinical Pharmacy, Zhengzhou University, Zhengzhou, China

**Keywords:** baicalin, sorafenib, bioavailability, drug–drug interactions, *in situ* single-pass rat intestinal perfusion model

## Abstract

**Background:** Sorafenib (SOR) is an oral, potent, selective, irreversible epidermal growth factor receptor tyrosine kinase inhibitor (EGFR-TKI) used as the first-line therapy for advanced hepatocellular carcinoma (HCC). Baicalin (BG) is used as adjuvant therapy for hepatitis, which accounts for the leading cause of the development of HCC, and is commonly coadministered with SOR in clinic. The purpose of the current study was to characterize the pharmacokinetic changes of SOR and the potential mechanism when SOR is administered concomitantly with BG in rats for single and multiple doses.

**Methods:** Parallel randomized pharmacokinetic studies were performed in rats which received SOR (50 mg/kg, *i.g.*) alone or coadministered with BG (160 mg/kg, *i.g.*) for single and multiple doses (7 days). Plasma SOR levels were quantified by ultra-performance liquid chromatography–tandem mass spectrometry (UPLC-MS/MS). Rat liver microsomes (RLMs) which isolated from their livers were analyzed for CYP3A and SOR metabolism activities. The inhibitory effect of BG on the metabolism of SOR was also assessed in pooled human liver microsomes (HLMs). The effects of BG on the intestine absorption behaviors of SOR were assessed in the *in situ* single-pass rat intestinal perfusion model.

**Results:** Coadministration with BG (160 mg/kg, *i.g.*) for single or multiple doses significantly increased the C_max_, AUC_0–t_, and AUC_0–∞_ of orally administered SOR by 1.68-, 1.73-, 1.70-fold and 2.02-, 1.65-, 1.66- fold in male rats and by 1.85-, 1.68-, 1.68-fold and 1.57-, 1.25-, 1.24- fold in female rats, respectively (*p* < 0.01 or *p* < 0.05). *In vitro* incubation assays demonstrated that there were no significant differences of *K*_*m*_, *V*_*max*_, and *CL*_*int*_ of 1-OH MDZ and SOR N-oxide in RLMs between control and multiple doses of BG-treated groups. BG has no obvious inhibitory effects on the metabolism of SOR in HLMs. In comparison with SOR alone, combining with BG significantly increased the permeability coefficient (*P*_*eff*_) and absorption rate constant (*K*_*a*_) of the SOR *in situ* single-pass rat intestinal perfusion model.

**Conclusion:** Notably enhanced oral bioavailability of SOR by combination with BG in rats may mainly account for BG-induced SOR absorption. A greater understanding of potential DDIs between BG and SOR in rats makes major contributions to clinical rational multidrug therapy in HCC patients. Clinical trials in humans and HCC patients need to be further confirmed in the subsequent study.

## 1 Introduction

Liver cancer ranks the third leading mortality and fifth most commonly occurring cancer among malignancies worldwide mainly due to lack of improvement therapies (Sung et al., 2021). Sorafenib (SOR), the first-generation multi-tyrosine kinase inhibitor for metastatic hepatocellular carcinoma (HCC) treatment, largely extends the overall survival rates of patients (Keating, 2017; Tang et al., 2020). A considerable amount of literature has indicated that SOR suppresses tumor proliferation, invasion, and metastasis by antagonizing the vascular endothelial growth factor receptor (VEGFR)– and platelet-derived growth factor receptor (PDGFR)–mediated classical pathways such as PI3K/AKT/mTOR and RAF/MEK/ERK signaling pathways (Saidak et al., 2017; Yokoi et al., 2018; Roderburg et al., 2020). However, the poor water solubility and high affinity to the multidrug transporters of SOR result in its limited bioavaliability (Raoul et al., 2019). In addition, the most commonly occurring adverse events associated with SOR treatment in HCC patients including rash, hypertension, and gastrointestinal bleeding lead to severe limitations for its clinical application (Park et al., 2020).

Baicalin (BG), the dominant active flavonoid compound extracted from *Scutellaria baicalensis* and the main bioactive constituent of the most frequently used traditional Chinese medicine, was approved for the adjuvant therapy of hepatitis (Huang et al., 2019). A growing body of evidence has indicated that BG exhibits various pharmacological activities, including antioxidant, antitumor, antimicrobial, antiapoptotic, neuroprotective, and anti-inflammatory activities (Hu et al., 2021). Recently, there has been an increasing amount of literature on that BG exerts potent antitumor effect on various cancers such as HCC, breast cancer, and lung cancer by targeting p38MAPK, Ras/Raf/MEK/ERK, and PKC/STAT3 signaling pathways and regulating cyclins and CDKs (Wang et al., 2018; Ke et al., 2019; Singh et al., 2021). Along with the growth in the clinical applicability of BG, there is increasing concern over the drug interactions with BG during coadministration with other agents (Li-Weber, 2009; Boniface and Elizabeth, 2019). Notably, for treatment of patients with complex disease states, BG exhibited synergistic interactions with many combination drugs by regulating drug-metabolizing enzymes and/or drug transporters to decrease/enhance their efficacy and reduce/increase toxicity in the complex therapeutic regimens (Kalapos-Kovacs et al., 2015; Kalapos-Kovacs et al., 2018; Zhou et al., 2021). We elucidated that BG varies the pharmacokinetics of phenacetin, theophylline, midazolam, dextromethorphan, nifedipine, and chlorzoxazone in rats by regulating the metabolism of CYP1A2, CYP2E1, CYP3A, and CYP2D in previous studies (Gao et al., 2013; Tian et al., 2013a; Tian et al., 2013b; Cheng et al., 2014; Gao et al., 2014). More recently, data from several sources have identified that BG could inhibit the expression of OATP1B1 to decrease the exposure of rosuvastatin in healthy volunteers and increase the absorption profiles to enhance the AUCs of geniposide in cerebral ischemia rats (Fan et al., 2008; Pan et al., 2013). Therefore, highlighting the potential DDIs and the underlying mechanisms between BG with combination drugs is essential for its clinical implications.

Recently, administration of combination therapies such as traditional herbal medicines to improve treatment effects of SOR has become a new therapeutic approach (Farzaei et al., 2016). The absorption of SOR was rapid and was mediated by several intestinal transporters in the gastrointestinal tract after oral administration (Gong et al., 2017). The metabolism of SOR in the liver is extensive, and the CYP3A4 (N-oxidation) and UGT1A9 (glucuronide conjugation) were the phase I and phase II enzymes, respectively (Gong et al., 2017; Xia et al., 2020). Hence, the transporters and metabolic enzyme–related drug interactions of SOR and coadministration drugs might occur. Xianming Wang et al. reported oral pretreatment of triptolide significantly increases the exposure of SOR in rats, which may account for the inhibition of CYP3A (Wang et al., 2017). Wang X et al. indicated coadministered with verapamil increased the AUC_0-t_ and C_max_ of SOR by 58.2 and 57.4%, respectively, in rats mediated by P-gp inhibition (Wang et al., 2016). David Paul revealed that treatment of palbociclib slightly increased the oral bioavailability of SOR in rats by inhibiting the metabolism (Paul et al., 2019). In addition, SoHyun Bae proved that 5, 7-dimethoxyflavone largely enhanced SOR AUC in plasma and most tissues in mice, and the mechanism mediated might be 5,7-DMF significantly increased the effluxion of SOR by inhibiting BCRP by the Bcrp1-dependent manner (Bae et al., 2018). As mentioned before, the unpredictable pharmacodynamic effects might be increasingly increased when SOR is coadministered with other therapeutic agents. Demonstrating the DDIs makes a major contribution to the clinical therapeutic implications of SOR.

To the best of our knowledge, both BG and SOR are effective against HCC, and BG is also used in adjuvant therapy for hepatitis (Hu et al., 2021). So there is a potential risk of coadministration in HCC patients who adopted BG and SOR for clinical therapeutic applications. However, to date, no studies regarding the drug interactions between SOR and BG have been reported. In the current study, we designed to gain an understanding of BG coadministration for single and multiple doses on the pharmacokinetic profiles of SOR in rats. The rat *in* situ single-pass intestinal perfusion model and the liver microsome incubation system would be implemented to uncover the potential regulatory mechanisms influencing the interactions. The study will provide guidance for dosage adjustment and rational multidrug therapy for SOR.

## 2 Materials and Methods

### 2.1 Chemicals and Reagents

Sorafenib (purity ≥99%) was purchased from MedChemExpress Co., Ltd. (Shanghai, China). Nilotinib (purity ≥99%) was procured from Aladdin Co., Ltd. (Shanghai). Baicalin (purity ≥98%) was supplied from Solarbio Industry (Beijing, China). MDZ injection was purchased from Nhwa Pharmaceutical Co., Ltd. (Xuzhou, China). Sorafenib N-oxide (purity ≥99%) was purchased from Toronto Research Chemicals Inc. (North York, Canada). 1-OH MDZ (purity ≥99%) was purchased from Sigma-Aldrich (St. Louis, MO). Urethane (purity ≥99%) was purchased from MedChemExpress Co., Ltd. (Shanghai, China). Human liver microsomes were purchased from the Research Institute for Liver Diseases Ltd Co (RILD) (Shanghai, China). NADPH was supplied from Solarbio Industry (Beijing, China). Formic acid (HPLC grade) was purchased from Aladdin Co., Ltd. (Shanghai). Ammonium acetate was purchased from Zhiyuan Chemical Co., Ltd. (Tianjin, China). Other liquid chromatography–grade reagents, including methanol and acetonitrile, were all obtained from Thermo Fisher Scientific (Fairlawn, NJ, United States). Ultrapure water was acquired from the Milli-Q water purification system (Millipore, Bedford, MA, United States).

### 2.2 Animals

The Sprague–Dawley rats weighing 230–250 g were purchased from the Beijing Vital River Laboratory Animal Technology Co., Ltd. (Beijing, China). Equal numbers of male and female rats were housed in different cages with free access to diet and water for acclimatization. The temperature was (25 ± 2°C), and the relative humidity was 50–60% with a 12-h light–dark cycle which housed the rats. The rats were fasted for 18 h but with free access to water prior to drug administrations. All experimental protocols were reviewed and approved by the Animal Ethics Committee of the First Affiliated Hospital of Zhengzhou University. In the case of animal care principles, we performed the animal facilities and welfare protocols rigorously in conformity to the National Institutes of Health guidelines.

### 2.3 *In vivo* Pharmacokinetic Experiments

A randomized and parallel experiment was designed to assess the influence of BG on the SOR pharmacokinetic profile in rats. SOR (50 mg/kg) was dissolved and suspended by 1% carboxymethylcellulose sodium solution. We dissolved 0.817 g BG in 50 ml of a Na_2_HPO_4_ solution (0.2 M) to prepare BG (160 mg/kg), and the mixture solution pH (pH = 7.40) was adjusted using citric acid (0.1 M).

To explore the effect of single dose of BG on the PK profile of SOR, we randomized divided rats into a control group and BG-treated group (*n* = 12 for each group and consist of equal numbers of male and female rats). One group received BG (160 mg/kg) and SOR (50 mg/kg), and another group received SOR (50 mg/kg). The rats coadministered with SOR and BG received oral BG 30 min prior to SOR.

For assessing coadministration with BG for multiple doses on the pharmacokinetic profile of SOR in rats, twenty-four rats with equal numbers of male and female were randomized and divided into the experimental groups (*n* = 12 for each) as follows: group I: the control group received normal saline for 7 days; group Ⅱ: orally administered with BG (160 mg/kg, *i.g.*) for consecutive 7 days. After successive 7 days, the rats in group I orally received SOR (50 mg/kg) on day 8. The rats in another group which were coadministered with BG for 7 days orally received SOR (50 mg/kg) with BG (160 mg/kg) on day 8, and BG was treated orally 30 min prior to SOR administration. We obtained blood samples (each 100 μl) from the ocular choroidal vein of rats into heparinized tubes at pre-dose and 0.25, 0.5, 1, 2, 4, 6, 8, 12, 24, 36, 48, and 72 h after the dose. The plasma samples of SOR in rats were centrifuged from blood samples at 4,000 rpm 4°C for 10 min and stored at −80°C until UPLC-MS/MS analysis. The developed UPLC-MS/MS method was adopted for the rat SOR plasma concentration determination.

### 2.4 Preparation of Rat Plasma Samples

Sample extraction from rat plasma was performed in a two-step protein precipitation method. To precipitate proteins, we mixed a 40 μl of the plasma sample in an aliquot, 5 μl of nilotinib solution (IS, 600 ng/ml), and 150 μl of protein precipitator ACN together. The mixture samples were mixed and vortexed for 1 min and centrifuged at 145,000 g for 10 min. We separated the supernatant of 100 μl from processed samples and transferred to fresh EP tubes. For quantification, we injected the supernatant solution of 3 μl into the UPLC-MS/MS system.

### 2.5 Ultra-Performance Liquid Chromatography–Tandem Mass Spectrometry Analysis

According to a published article regarding the LC-MS/MS method, we analyzed and determined the SOR concentration in rat plasma with minor modifications (Allard et al., 2017; Iacuzzi et al., 2020). The Qtrap 4500 mass spectrometer equipped with a turbo ionspray interface connected to the ExionLC analytical system (AB Sciex, United States) was adopted for analysis.

Chromatographic separation of SOR was performed on a Phenomenex Kinetex C18 column (2.1 × 50 mm, 2.6 μM) with the column temperature maintained at 40°C. The mobile phase system composed of water (containing 2 mM ammonium acetate aqueous solution and 0.1% formic acid) and acetonitrile (25:75, v:v). The flow rate was 0.4 ml/min, and the total run time for separation was within 3 min. The retention times for SOR and nilotinib were 0.86 and 0.71 min, respectively.

In the aspect of MS/MS detection, a multiple reaction monitoring (MRM) model was carried out, and an electrospray ionization source (ESI) in positive mode was selected accounting for both SOR and IS exhibited the strong responses. In the view of full scan mass spectrum data, the transition ions for detection were from m/z 465.0–252.0 for SOR and m/z 530.1–289.1 for IS, respectively. The MS/MS condition for quantification was given as follows: source temperature 500°C, ion spray voltage 5500 V, nebulizer gas (gas 1) 50 psi, and heater gas (gas 2) 50 psi. The dwell time for SOR and IS was 100 ms. We performed the data acquisition with Analyst™ software (AB Sciex, version 1.6.3, United States).

### 2.6 Measurement of CYP3A and Sorafenib Metabolism Activity in Rat Liver Microsomes

CYP3A activity was assessed by the formation of 1-OH MDZ in RLMs. In terms of the *in vitro* incubation system, we mixed 10 μl of MDZ, 10 μl of rat liver microsomal protein (0.2 mg/L), 10 μl of NADPH (10 mM), 50 μl of sodium phosphate buffer (0.1 M, pH 7.4), and 20 μl of water together in a final total volume of 100 μl. The *in vitro* incubation mixture was preincubated at 37°C for 5 min and then NADPH was added to initiate the process. We performed the whole incubation process at 37°C for 10 min and terminated by appending ice-cold acetonitrile containing IS (10 ng/ml). To assess the effect of BG on the metabolism of SOR in RLMs, the *in vitro* incubation system was also carried out. The entire *in vitro* incubation mixture was performed as follows with the final total volume of 100 μl: SOR (10 μl), 0.4 mg/L rat liver microsomal protein (10 μl), 0.1 M sodium phosphate buffer (50 μl, adjusting pH to 7.4), and water (20 μl). We preincubated the *in vitro* incubation mixture at 37°C for 5 min and started the reaction by accretion of NADPH. The total incubation period time for this reaction was 20 min at 37°C, and the termination process was performed by adding 20 μl of ice-cold acetonitrile including IS (10 ng/ml). After vortexing the mixture for 1 min, the supernatants were achieved from the processed samples which were centrifuged at 12,000 rpm for 10 min at 4°C. Afterward, we injected the supernatants into UPLC-MS/MS for analysis.

### 2.7 Effects of Baicalin on the Metabolism of Sorafenib in Human Liver Microsomes

The pooled human liver microsome (HLM) incubation system was selected for evaluating the effects of BG on the metabolic activity of SOR. We performed incubation reaction mixtures in conformity to the literature published recently with few modifications (Burns et al., 2015). The incubation system with a total volume of 100 μl comprises BG with different concentrations (10 μl), SOR (10 μl), 10 μl HLMs protein (0.2 mg/L), 10 μl NADPH (10 mM), 50 μl sodium phosphate buffer (0.1 M, adjusting pH to the level of 7.4), and water (10 μl). For the purpose of investigating the inhibitory effects of BG on the metabolism of SOR, BG (0.1, 1, 2, 5, 10, 20, 50, and 100 μM) was mixed with HLMs for preincubating in advance at 37°C for 15 min. Afterward, NADPH was added for activating the reaction until the whole incubation mixture preincubated for 5 min at 37°C. After the period of the reaction for 20 min, 20 μl ice-cold acetonitrile composed of IS (10 ng/ml) was added to the incubation mixture for terminating the reaction. Then we vortexed the mixture for 1 min and centrifuged for 10 min at 12,000 rpm at 4°C. The supernatants were obtained from the processed samples and injected into UPLC-MS/MS system for further analysis.

### 2.8 Effects of Baicalin on the Absorption of Sorafenib in the *in situ* Single-Pass Rat Intestinal Perfusion Model

The Krebs–Ringer’s (K-R) nutrient solution contained 195 mM NaCl, 3.22 mM CaCl_2_ (resolved independently in advance of adding to the entire solution), 0.21 mM MgCl_2_, 2.67 mM NaH_2_PO_4_, 16.3 mM NaHCO_3_, and 7.06 mM D-glucose. After mixing all solutions together, 0.1 M HCl solution was added for accommodating the pH of the solution to 7.4.

According to the procedure of the article published previously, we established the *in situ* single-pass intestinal perfusion model with slight modifications (Roos et al., 2017). Prior to the study, we starved 12 male rats which weighed 200–220 g for 12 h but with free access to water. Then, they were narcotized by administering a peritoneal injection with urethane at a dose of 0.3 ml/100 g. The narcotized rats were operated on a fixing plate in a supine position. To maintain the normal body temperature (37°C), the anesthetized rats were kept under a heating lamp during the experiments. The jejunum (10 cm) was isolated from the intestinal segments after opening the abdominal cavity along the midline of the abdomen. After interposing two silicone tubes attentively to either ends of the jejunum through the small narrow opening, we ligated them with a sterile surgical line. The K-R nutrient solution was preheated to 37°C in advance. Afterward, the intestinal contents were rinsed completely with addition of the preheated K-R nutrient solution. The remaining K-R solution was drained by pumping air into the intestines. The intestines were returned to the abdominal cavity to maintain their integrity until the effluent solution was free of feces and clear. After the surgery, the intestines were perfused at a flow rate of 1 ml/min for 5 min and balanced at a flow rate of 0.2 ml/min and maintained for approximately 30 min with the K-R nutrient solution.

In order to probe into the BG treatment on the absorptive profile of SOR, the K-R nutrient solution containing SOR and the K-R nutrient solution containing SOR and BG (2, 5, and 10 μM) were, respectively, perfused into the intestinal segments immediately from the peristaltic pump inlet. Afterward, the tiny bottles which contained the perfusion solution and collected outflow perfusate had been accurately weighed. Then the perfusate samples from the perfused intestinal segments were collected and weighed every 15 min (15, 30, 45, 60, 75, 90, 105, and 120 min). The collected perfusate samples were transferred into 2-mL Eppendorf tubes and centrifuged at 5,000 rpm for 15 min. The supernatant from the processed samples with a total volume of 200 μl was transferred and diluted to be analyzed by UPLC-MS/MS on the same day. At the end, the corresponding intestinal segments were dissected respectively and their length (L) and perimeter (s) recorded.

In the *in situ* single-pass rat intestinal perfusion model, the *P*_*eff*_, which represents the effective permeability coefficient (cm/s), was calculated using the following equation.

$$P_{eff}=\;\frac{-Q\times In\left(C_{out}/C_{in}\right)}{2\pi RL}.$$

*K*_*a*_, which represents the absorption rate constant, was calculated using the following equation:

$$K_{a}=\frac{Q\left(C_{in}-C_{out}\right)}{C_{in}\times \pi R^{2}L}\times 100\%,$$

where *Q* is the flow rate at 0.2 ml/min, *C*_*out*_ is the intestinal luminal drug concentration collected after perfusion at time t, *C*_*in*_ is the initial perfused drug concentration preperfusion, and *R* and L (cm) are the radius and the length of the intestinal segment, respectively.

### 2.9 Pharmacokinetic Analysis

Pharmacokinetic parameters of SOR in rats were evaluated and calculated in the noncompartmental model by Phoenix WinNonlin (version 8.1, SCI software, Statistical Consulting, Inc., Apex, NC, United States). The maximum concentration (C_max_) and the time to C_max_ (T_max_) were observed and acquired from experimental data. The linear trapezoidal method was adopted to calculate the area under the curve to the last measurable concentration (AUC_0–t_). The half-life (t_1/2_) was estimated from elimination constants, and the clearance (CL) was evaluated as Dose/AUC_0-inf_.

### 2.10 Statistical Analysis

SPSS 11.5 (LEAD Technologies Inc.) was used for statistical evaluation. The statistical significance differences of mean values in multiple groups were analyzed using one-way ANOVA followed by Tukey's post hoc test, and Student’s *t*-test was used for two group comparisons. It is considered to be statistically significant when *p* values are less than 0.05 (*p* <0.05).

## 3 Results

### 3.1 Effects of Baicalin on the Pharmacokinetics of Sorafenib in Rats

#### 3.1.1 Effects of the Single Dose of Baicalin on the Pharmacokinetics of Sorafenib in Rats

**FIGURE 1:**
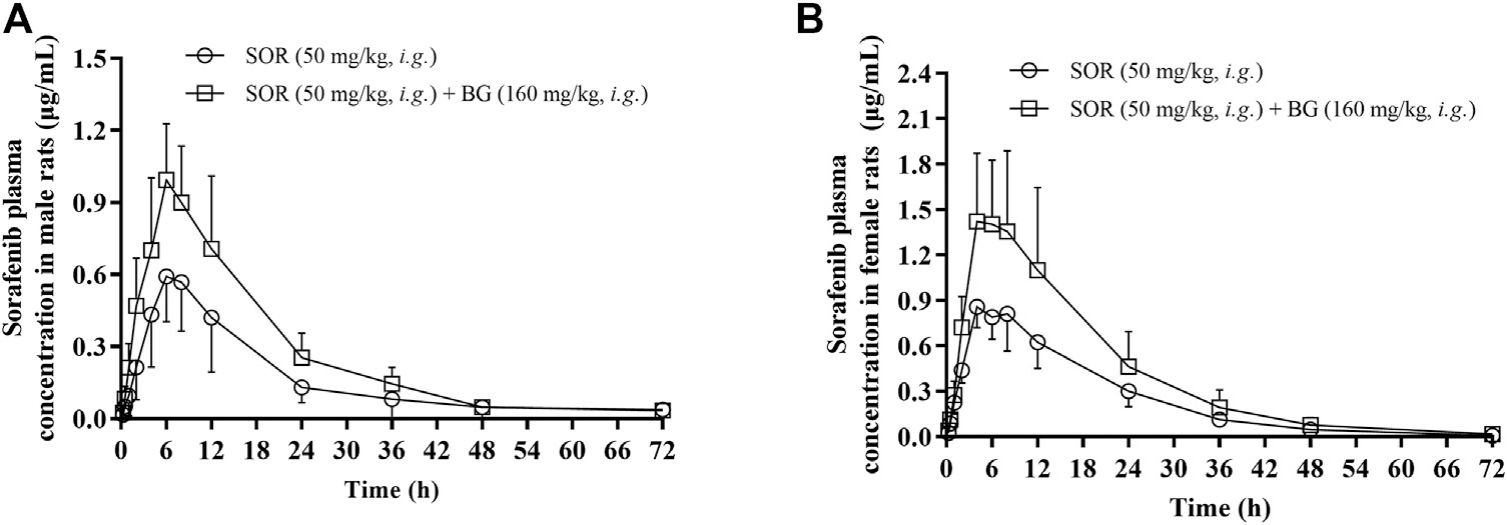
Mean plasma concentration–time profiles of SOR in male **(A)** and female **(B)** rats following oral administration of SOR (50 mg/kg, *i.g.*) with a single dose of BG (160 mg/kg, *i.g.*). Data are presented as mean ± SD. Six rats were used for each data point in both control and BG treatment groups (*n* = 6).

The mean plasma concentration–time curves after the oral administration of SOR (50 mg/kg, *i.g.*) for a single dose and in combined dosing BG (160 mg/kg, *i.g.*) were demonstrated in Figure 1. PK parameters were estimated and tabulated in Table 1. There was a significant difference in the exposure of SOR between male rats and female rats, and the exposure of SOR in female rats was much higher than that in male rats.

**TABLE 1:** Sorafenib pharmacokinetic parameters in male and female rats after oral administration of SOR (50 mg/kg) alone and concomitant treatment with or without of BG (160 mg/kg, *i.g.*) (*n* = 6, each).

PK parameter	Male		Female	
	Control	BG + SOR	Control	BG + SOR
C_max_ (μg/ml)	0.60 ± 0.19	1.01 ± 0.23**	0.92 ± 0.15^##^	1.70 ± 0.35**
t_max_ (h)	6.33 ± 1.50	6.00 ± 1.27	5.33 ± 2.07	6.00 ± 1.79
t_1/2_ (h)	18.18 ± 8.14	16.15 ± 6.61	10.75 ± 6.09	8.79 ± 2.51
AUC_0∼t_ (h·μg/ml)	10.82 ± 5.07	18.74 ± 4.67*	17.40 ± 4.19^##^	29.25 ± 12.03*
AUC_0∼∞_ (h·μg/ml)	11.48 ± 5.81	19.51 ± 4.54**	17.59 ± 4.51^##^	29.57 ± 12.32*
MRT_0∼t_ (h)	16.13 ± 2.60	17.08 ± 3.39	15.67 ± 1.12	15.26 ± 5.54
MRT_0∼∞_(h)	19.70 ± 3.44	20.427 ± 5.17	16.58 ± 0.92	15.86 ± 2.84
CL/F (L/h)	5.27 ± 2.36	2.68 ± 0.62*	3.02 ± 0.83^##^	1.91 ± 0.65*
Vz/F (L)	147.37 ± 100.14	66.50 ± 42.08	51.00 ± 41.96	22.43 ± 5.05

Values are means ± SD.

*p < 0.05, **p < 0.01 compared with the control.

^##^ *p* < 0.01 compared with the male group.

The results showed that a single dose of BG increased the C_max_, AUC_0-t_ and AUC_0-∞_ of SOR in male and female rats significantly and decreased the oral clearance rate of SOR notably. In female rats, the C_max_, AUC_0–t_, and AUC_0–∞_ of SOR combined with BG were 1.85, 1.68, and 1.68 times, respectively, higher than those of SOR alone. For male rats, compared to the rats administered with SOR alone, coadministration of BG significantly enhanced C_max_, AUC_0–t_, and AUC_0–∞_ by 1.68, 1.73, and 1.70-fold, respectively, and decreased CL by 49%. No significant difference of t_1/2_ was observed between the groups.

#### 3.1.2 Effects of Multiple Doses of Baicalin on the Pharmacokinetics of Sorafenib in Rats

**FIGURE 2:**
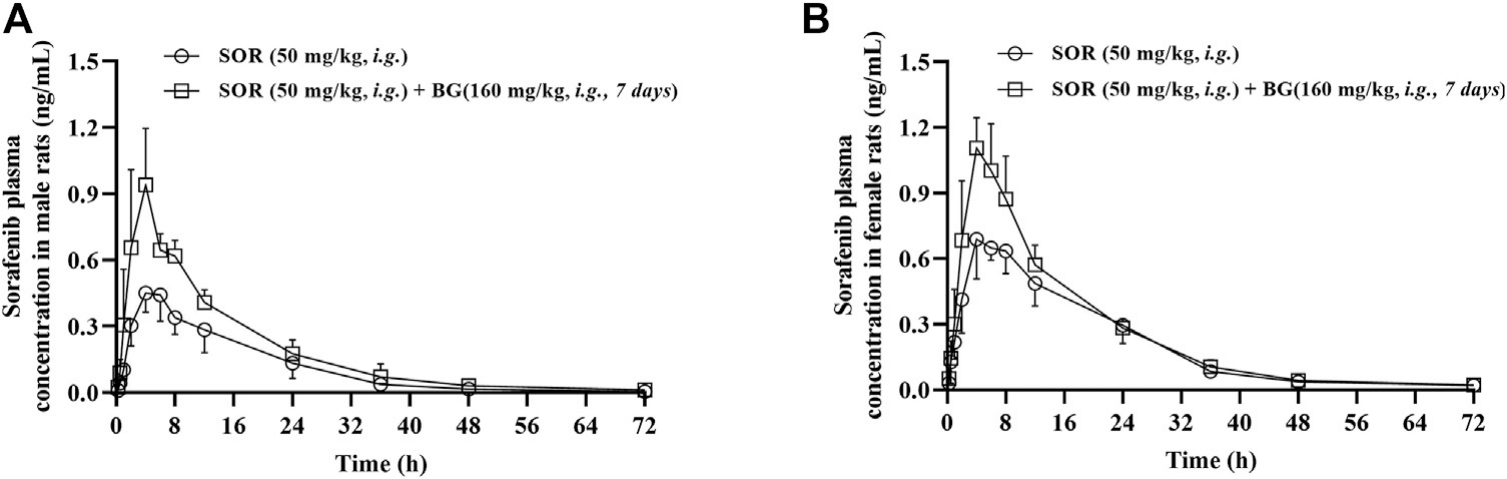
Mean plasma concentration–time profiles of SOR in male **(A)** and female **(B)** rats following SOR administration (50 mg/kg, *i.g.*) orally together with multiple doses of BG (160 mg/kg, *i.g*) for seven consecutive days. Data are presented as mean ± SD. Six rats were used for each data point in both control and BG treatment groups (*n* = 6).

The mean plasma concentration–time curves of SOR in the control group and multiple doses groups (160 mg/kg, 7 days, *i.g.*) are shown in Figure 2. The major pharmacokinetic parameters of SOR are presented in Table 2. The C_max_ and AUC of SOR in female rats were significantly higher than those in male rats (*p* < 0.05).

**TABLE 2:** Sorafenib pharmacokinetic parameters in male and female rats after oral administration of SOR (50 mg/kg) alone and concomitant treatment with or without of BG (160 mg/kg, *i.g.*) for consecutive 7 days (*n* = 6, each).

PK parameter	Male		Female	
	Control	BG + SOR	Control	BG + SOR
C_max_ (μg/ml)	0.49 ± 0.06	0.99 ± 0.25**	0.77 ± 0.11^##^	1.21 ± 0.08**
*t*_*max*_ (h)	4.33 ± 1.51	4.33 ± 1.97	5.00 ± 1.67	5.21 ± 1.09
*t*_*1/2*_ (h)	9.21 ± 2.38	13.29 ± 5.48	11.84 ± 2.28	12.76 ± 3.16
AUC_0∼t_ (h·μg/ml)	7.97 ± 2.62	13.17 ± 2.00**	14.77 ± 1.39^##^	18.43 ± 1.96**
AUC_0∼∞_ (h·μg/ml)	8.08 ± 2.67	13.44 ± 2.16**	15.15 ± 1.57^##^	18.83 ± 2.10**
MRT_0∼t_ (h)	13.67 ± 3.08	14.07 ± 2.60	16.67 ± 1.50	15.27 ± 0.94
MRT_0∼∞_ (h)	14.47 ± 3.38	15.54 ± 3.06	18.46 ± 2.19	16.85 ± 1.18
CL/F (L/h)	6.95 ± 2.82	3.81 ± 0.63*	3.33 ± 0.34^##^	2.68 ± 0.30**
Vz/F (L)	88.27 ± 34.75	69.92 ± 22.71	56.73 ± 11.15	49.29 ± 13.71

Values are means ± SD.

*p < 0.05, **p < 0.01 compared with the control.

^##^ *p* < 0.01 compared with the male group.

As depicted in Table 2, in female rats, in comparison with the control group, coadministered BG at 160 mg/kg caused a notable increase in AUC_0–∞_ from 15.15 ± 1.57 to 18.83 ± 2.10 h μg/ml, and a significant increase in AUC_0–t_ from 14.77 ± 1.39 to 18.43 ± 1.96 h μg/ml, and a significant increase in C_max_ from 0.77 ± 0.11 to 1.21 ± 0.08 μg/ml and a significant decrease in CL/F from 3.33 ± 0.34 to 2.68 ± 0.30 L/h/kg, respectively (*p* < 0.01). As illustrated in Table 2, in male rats, BG significantly increased SOR C_max_, AUC_0-t_, and AUC_0-∞_ by 2.02-, 1.65-, and 1.66-fold, respectively (*p* < 0.01). When BG was coadministered with 160 mg/kg SOR, the CL/F was decreased 1.82-fold (*p* < 0.05). BG has no influence on the t_1/2_ of SOR both in female and male rats.

### 3.2 CYP3A and Sorafenib Metabolism Activity in Rat Liver Microsomes

**FIGURE 3:**
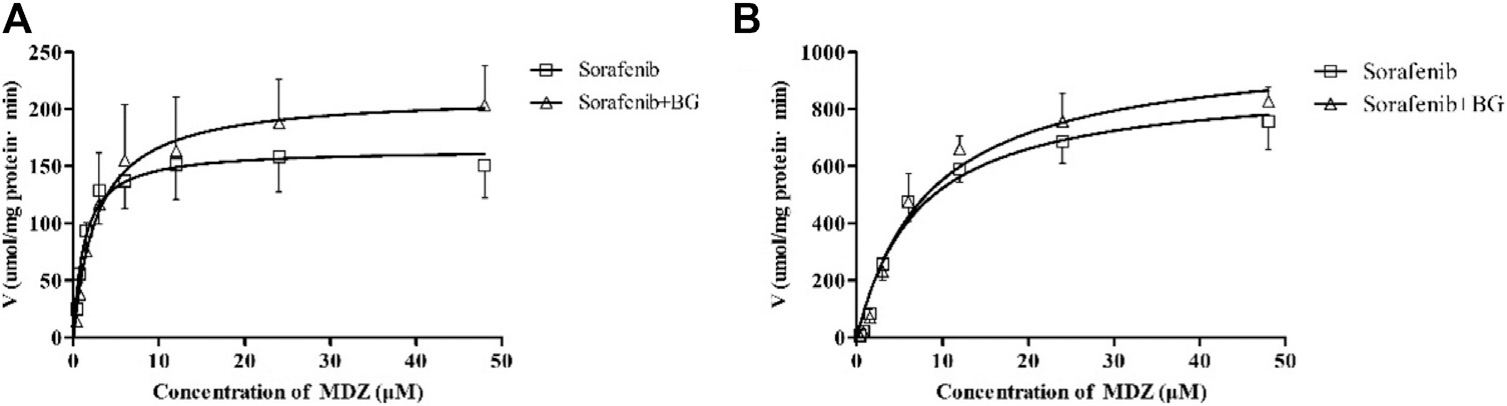
Kinetics of formation of 1-OH MDZ in female **(A)** and male **(B)** rat liver microsomes. Data represent the means ± SD of six independent experiments in duplicate determinations.

1-OH MDZ (the metabolite of MDZ) generation was determined in control and multiple doses of BG groups for determination of CYP3A activity. Figure 3 depicts the CYP3A activity in RLMs in control and BG groups. The related enzymatic kinetic parameters are described in Table 3. Male rats showed a much higher *V*_*max*_ and *K*_*m*_ values compared with female rats, which was in accordance with the pharmacokinetic studies. However, no significant difference was observed in the CYP3A-mediated MDZ hydroxylation activity between the BG group (160 mg/kg, 7 days) and the control group.

**TABLE 3:** Effects of multiple doses of BG (160 mg/kg, *i.g.*) for consecutive 7 days on MDZ hydroxylation activity in rat liver microsomes (*n* = 6).

Parameter	Female		Male	
	Control	BG	Control	BG
V_max_ (μmol/min/mgprotein)	164.70 ± 33.08	215.07 ± 4.87	899.73 ± 94.13*	1,026.20 ± 111.98
K_m_ (μM)	1.26 ± 0.23	2.10 ± 0.57	7.31 ± 1.53*	8.75 ± 2.38
CL_in_ (μl/min/mg protein)	132.14 ± 26.53	119.47 ± 36.91	125.97 ± 24.50	122.50 ± 31.15

Values are means ± SD.

*p < 0.05, compared with the female group.

**FIGURE 4:**
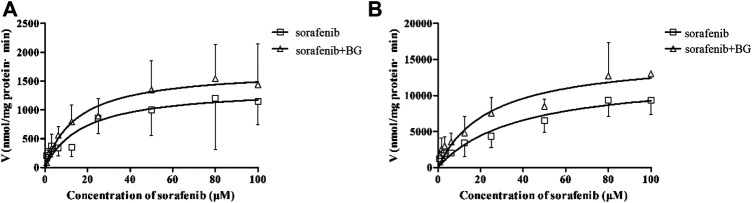
Kinetics of formation of Sorafenib N-oxide in female **(A)** and male **(B)** liver microsomes. Data represent the means ± SD of six independent experiments in duplicate determinations.

CYP3A was not the unique enzyme for the SOR metabolism. We further explored the SOR metabolism activity in RLMs. The SOR metabolism activity in RLMs is illustrated in Figure 4, and kinetic parameters are described in Table 4. The metabolism of SOR in male rats was much higher than that in the female rats. Similar to MDZ metabolism, there were no significant differences of *K*_*m*_, *V*_*max*_, and *CL*_*int*_ between control and multiple doses of the BG group.

**TABLE 4:** Effects of multiple doses of BG (160 mg/kg, *i.g.*) for consecutive 7 days on sorafenib N-oxide activity in rat liver microsomes (*n* = 6).

Parameter	Female		Male	
	Control	BG	Control	BG
V_max_ (nmol/min/mgprotein)	1,405.2 ± 782.3	1,714.13 ± 749.52	12,705.3.2 ± 2,379.8*	16,176.5 ± 976.51
K_m_ (μM)	17.42 ± 5.44	14.24 ± 6.36	37.87 ± 8.30*	31.46 ± 23.01
CL_in_ (μl/min/mg protein)	76.73 ± 20.63	121.23 ± 26.90	344.0 ± 89.16*	686.44 ± 471.01

Rat values are means ± SD.

*p < 0.05, compared with the female group.

### 3.3 Effects of Baicalin on the Metabolism of Sorafenib in Human Liver Microsomes

**FIGURE 5:**
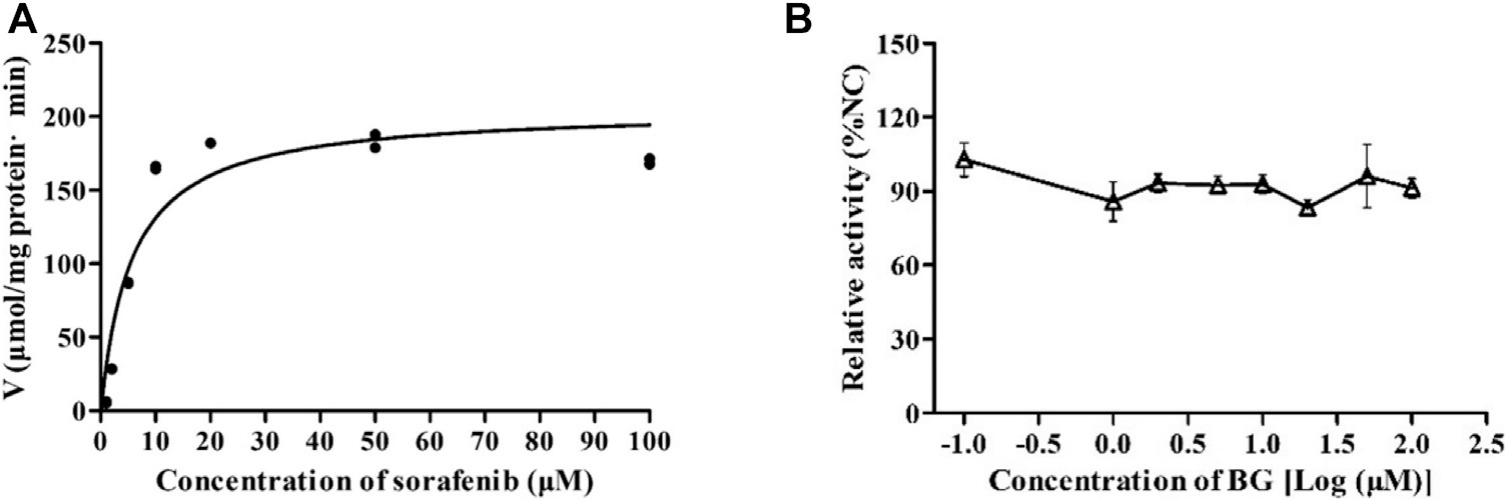
Kinetics of formation of sorafenib N-oxide in pooled human liver microsomes **(A)** and the effect of BG on the metabolic activity of SOR in pooled HLMs **(B)**. Data represent the means ± SD of three independent experiments in duplicate determinations.

The inhibitory effects of BG on the metabolism of SOR were investigated in HLMs. In Figure 5A, the *K*_*m*_, *V*_*max*_, and *CL*_*int*_ of SOR in HLMs were 5.66 μM, 205.2 μmol/min/mg protein, and 36.25 μl/min/mg protein in the absence of BG. The inhibition assays revealed the IC_50_ estimated large than 100 μM. It has been suggested that BG has no obvious inhibitory effects on SOR in HLMs (Figure 5B).

### 3.4 Effects of Baicalin on the Absorption of Sorafenib *in situ Single-Pass* Rat Intestinal Perfusion Model

**FIGURE 6:**
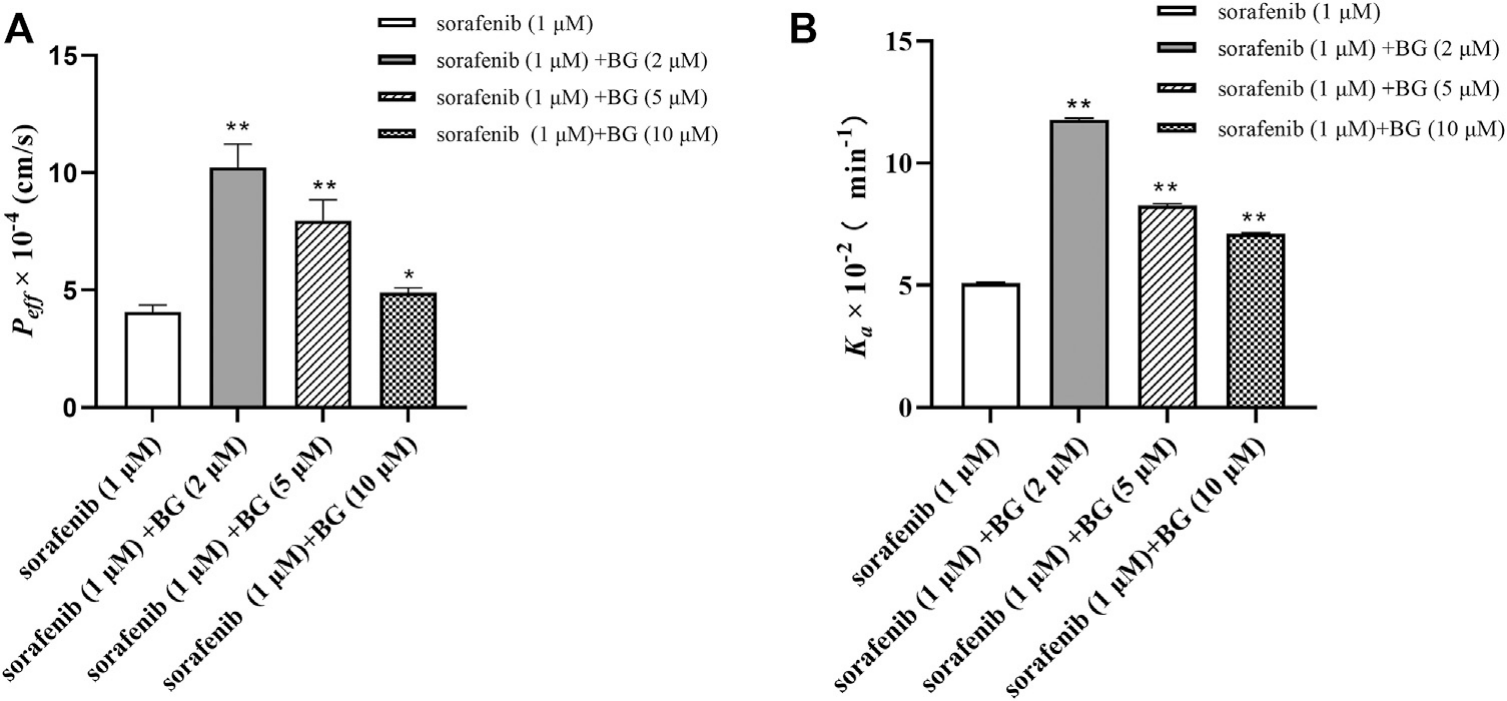
*P*_*eff*_ **(A)** and *K*_*a*_ **(B)** of SOR *in situ* single-pass rat intestinal perfusion experiments. Data represent the means ± SD of three independent experiments in duplicate determinations. Differences significantly compared with control: *p < 0.05, **p < 0.01.

The effect of BG on the *P*_*eff*_ and *K*_*a*_ of SOR is shown in Figure 6. The *P*_*eff*_ were 4.07 ± 0.28, 10.23 ± 0.99, 7.97 ± 0.88, and 4.89 ± 0.21 cm/s (10^−4^) in the control, BG (2 μM), BG (5 μM), and BG (10 μM). And SOR had Ka values of 5.10 ± 0.03, 11.78 ± 0.07, 8.28 ± 0.06, and 7.11 ± 0.03 min^−1^ (10^−2^) in the different groups. Compared to the control group, the *P*_*eff*_ of SOR increased 2.51, 1.96, and 1.20-fold and *K*_*a*_ of SOR increased 2.31, 1.62, and 1.39-fold in the intestine. The *P*_*eff*_ and *K*_*a*_ values of SOR increased when coadministered with BG. The results indicated that BG could significantly increase *P*_*eff*_ and *K*_*a*_ of SOR in the *in situ* single-pass rat intestinal perfusion model. Results indicated that BG could increase the absorptive rate of SOR *in situ* single-pass rat intestinal perfusion model. The results further revealed that BG could enhance the intestinal absorption of SOR in rats.

## 4 Discussion

The routine conventional chemotherapy in HCC patients which requires polypharmacy frequently including alternative medicaments and herbs largely augments the risk of DDIs (Yeung et al., 2018). Since BG and many compound preparations including active ingredient BG are widely used in the adjuvant therapy for hepatitis and found to exert anti-inflammatory and antiviral effects, many HCC patients were increasingly being administered with BG as they were frequently accompanied by variable degrees of chronic hepatitis, virus infection, and cirrhosis (Dinda et al., 2017; Hu et al., 2021). For these reasons, there are many cases where BG and SOR could and should be used concomitantly. There have been a large amount numbers of longitudinal studies uncovered that phenytoin, phenobarbital, and rifampin could alter the pharmacokinetic/pharmacodynamic profiles of *SOR via* inducing CYP3A4 (Fogli et al., 2020). And, it has conclusively been shown that SOR exhibited pharmacokinetic interactions with irinotecan, docetaxel, etc. for clinical practice (Awada et al., 2012; Fogli et al., 2020; Meany et al., 2021). In view of all that has been published articles so far, there is lack of study demonstrating the DDIs and the precise mechanism between BG and SOR in rats. Therefore, this study aimed to demonstrate coadministration with BG for single and multiple doses in rats on the pharmacokinetics of SOR and the potential mechanism for the first time.

In clinic, the patients were administered with an oral daily dosing of SOR 400–800 mg for the treatment of HCC and coadminisered with BG for 500–1,500 mg daily. According to the body surface area (BSA), the dosage which this study adopted for SOR was 50 mg/kg and BG was 160 mg/kg in rats (Blanchard and Smoliga, 2015). The experimental animals were all given an administration dosage which is equal to the clinical equivalent dosage in HCC patients. The maximal plasma concentrations levels (C_max_) of SOR in rats were approximately 2.82–6.67 h after oral administration SOR (50 mg/kg) for single dose. The t_1/2_ was approximately 6.83–14.12 h with a gradual decrease process. The pharmacokinetic properties of SOR in rats in our present findings seem to be consistent with other studies (Paul et al., 2019; Karbownik et al., 2020). In addition, what is interesting in these data is that the pharmacokinetic profiles of SOR have been showed marked gender-specific differences. The clearance of SOR was much slower in female rats than that in the male rats, contributing to notably higher C_max_ and AUC_s_ in female rats. These findings will guide the dose optimization for clinical settings.

Coadministered with BG (160 mg/kg, *i.g.*) for single and multiple doses increased the plasma exposure of SOR in female and male rats. The increase in C_max_ and AUC_s_ and the decrease CL/F of SOR were observed in rats for coadministration with BG. It is therefore conceivably hypothesized that BG inhibited the metabolism of SOR and/or increased the absorption of SOR. Our previous results demonstrated that intravenous injection with BG for consecutive 7 days at a dose of 0.90 g/kg enhanced the AUC_0–∞_ of midazolam in rats, and the C_max_ and AUC of dextromethorphan increased by 50 and 16% in rats following BG (0.90 g/kg, *i.v.*, 12 days) (Tian et al., 2013a; Tian et al., 2013b). The mechanism mediated might be multiple doses of BG-inhibited CYP3A in a non-competitive manner, and the K_i_ value was 60.8 μM. Besides, our recent results showed the oral bioavailability of CsA notably decreased when coadministered with BG orally (80 mg/kg) for consecutive 7 days, and the mechanism that uncovers this interaction might be BG induction of the P-GP–mediated absorption of CsA in the intestine (Tian et al., 2019). And the CYP3A expression exhibited no significant changes in oral multiple doses of the BG-treated liver compared with the control group in our recent studies (Tian et al., 2019). It is speculated that different CYP3A substrates showed different specificities, accounting for BG competitively displaced the plasma proteins of nifedipine to decrease its exposure in rats (Cheng et al., 2014).

It is possible to hypothesize that BG inhibited the metabolism of SOR, causing the increased exposure of SOR in rats coadministration with BG. Recent evidence suggests that SOR undergoes CYP3A4 metabolism in the liver; it is speculated that CYP3A inhibition by BG contributed to this interaction. To validate this proposition, we evaluated the CYP3A activity in RLMs with multiple doses of BG. The CYP3A activity was measured by MDZ (CYP3A substrate) clearance. The results indicated BG (160 mg/kg, *i.g.*, 7 days) treatment has no significant influence on *K*_*m*_, *V*_*max*_, and *CL*_*int*_ of 1-OH MDZ. These results demonstrated BG has no obvious effect on CYP3A in rats. The present findings seem to be consistent with those of Yue Li et al. who reported that no significant differences of CYP3A activity were observed with BG treatment in LS174T cells (Li et al., 2010). However, the literature has emerged that offers contradictory findings about BG treatment for 24 and 36 h inducing the expression of CYP3A and BG incubation for 48 h inhibiting the CYP3A expression in Chang liver cells (Fan et al., 2009). This rather contradictory result may be due to that the experiment materials were different. Apart from CYP3A, the metabolism of SOR by glucuronide conjugation is mainly mediated by UGT1A9 (Gong et al., 2017). We further evaluated the SOR metabolism in RLMs. Similarly, there were no significant differences of the *K*_*m*_, *V*_*max*_, and *CL*_*int*_ of sorafenib N-oxide in the BG-treated group in comparison with those of the control group in RLMs.

These results demonstrated that BG may have no influence on the hepatic metabolism of SOR in rats. Our previous finding that BG non-competitively inhibited CYP3A has been inconsistent with our present results. A reasonable explanation to tackle this issue might be the notably lower BG dosage adopted in the present study, and the distribution of BG in the liver could not exceed over the inhibition constant (K_i_ value) (Tian et al., 2013a; Tian et al., 2013b). Furthermore, we also investigated BG treatment for different concentrations on the metabolism of SOR in HLMs, and the results would seem to suggest that the IC_50_ estimated is large than 100 μM. It has been suggested that BG has no obvious inhibitory effects on SOR metabolism in HLMs. In accordance with the present results, our recent study revealed that the CYP3A expression was not influenced by BG treatment for oral multiple doses (Tian et al., 2019). As mentioned before, metabolism inhibition may not be the major reason for increased exposure of SOR in rats following multiple doses of BG.

Apart from metabolism, the absorption of the drug also related to oral bioavailability (Xue et al., 2019). Our hypothesis about the oral bioavailability of SOR increment in rats by coadministration with BG is largely based on absorption enhancement, for the reason of the t_1/2_ of SOR in rats has been observed with no significant changes. Therefore, it is possible to hypothesize that BG induced the intestine absorption of SOR in rats. Oral absorption profiles of SOR in rats could be estimated in various *in vitro* and *in situ* models such as the Caco-2 cell monolayer model, everted gut sac model, intestinal perfusion model, and the rat single-pass intestinal perfusion model (Alqahtani et al., 2013). It is considered that the *in situ* single-pass intestinal perfusion model is the best model among these models adopted for its full blood supply and drug-metabolizing enzyme and transporters expressed. Therefore, we examined the absorptive profiles of SOR with/without the treatment of BG in the *in situ* single-pass rat intestinal perfusion model. The *P*_*eff*_ and *K*_*a*_ of the drugs are the key biopharmaceutical variables to access the rate and extent of intestinal absorption (Yim et al., 2020). In the present study, coperfusion of SOR with BG demonstrated significantly higher *P*_*eff*_ and *K*_*a*_ values in the intestine in the studied concentration range. The results indicated BG could enhance the absorption rates of SOR in the intestine. The intestinal absorption of SOR induced by BG may be the reason for the increased exposure of SOR in rats. Therefore, we hypothesized that the intestinal absorptive differences in different groups may be caused by the different activities of transporters mediated by BG.

The intestinal transporters, including the ATP-binding cassette (ABC) superfamilies of efflux transporters and the solute carrier (SLC) superfamilies of cellular influx and efflux carriers, display a vital role in the absorption of SOR (Edginton et al., 2016; Wang et al., 2016). The literature indicated SOR has been found to be a substrate of the organic anion transporting polypeptide (OATP) and organic cation transporter-1 (OCT1) (Zimmerman et al., 2013; Grimm et al., 2016). SOR also showed moderate affinity for P-glycoprotein (P-gp) and breast cancer resistance protein (BCRP) (Agarwal and Elmquist, 2012). Our recent results demonstrated the P-gp expression enhanced notably in the intestine of rats for oral treatment of BG at a dose of 80 mg/kg for consecutive 7 days (Tian et al., 2019). So, P-gp induction by multiple doses of BG may account for the increasing extent of AUCs of SOR in rats, which was lower in multiple doses of BG group than in the single dose of BG group. Chung-Ping Yu et al. demonstrated Scutellariae radix (which contains plenty of flavonoids such as baicalin) could inhibit the BCRP- and MRP2-mediated efflux transports in MDCKII-BCRP cells (Yu et al., 2016). And Bernadett Kalapos-Kovács et al. indicated BG inhibited BCRP-mediated transport with an IC_50_ of 3.41 ± 1.83 μM in mammalian cells (Kalapos-Kovacs et al., 2015). Several attempts have been made to prove that BG notably inhibited the activities of OATs and significantly decrease the influx of OATs substrates (Xu et al., 2013). A number of researchers have sought to determine that oral administration of BG rapidly converted to baicalin (B) on the intestinal microflora and B dominantly metabolized to BG in the liver (Baradaran Rahimi et al., 2021; Hu et al., 2021). Peng Xu et al. reported B could alter the pharmacokinetic and pharmacodynamic profiles of silybin significantly by inhibition of BCRP and MRP2 (Xu et al., 2018). And Tatsuya Kawasaki et al. reported B inhibited OATP2B1 notably in OATP2B1-overexpressed HEK293 cells (Kawasaki et al., 2020). We speculated that BG and B, which exist in the intestine, mainly modulate the activity and expression of transporters and enhance the oral bioavailability of SOR in rats.

In reviewing the literature, this is the first study to undertake a systematic analysis of the pharmacokinetic drug-interactions between BG and SOR in rats. In the current study, our finding confirmed coadministration with BG (160 mg/kg, *i.g.*, 7 days) significantly enhanced the oral bioavailability of SOR in rats, and the mechanism might be BG induced by the absorption of SOR in the intestine. These interaction studies should be evaluated in healthy volunteers and HCC patients in the future for the large species differences in regarding to the absorption and metabolism of SOR. As BG and SOR co-administration is safe, well tolerated in rats, and both agents suppress HCC, it is considerable to have been the potential that BG enhanced the pharmacodynamic effects of SOR for HCC therapy (Wang et al., 2018; Ke et al., 2019; Singh et al., 2021). However, care should be paid for BG and SOR co-administration therapy as the enhanced adverse effects also might be increasingly recognized as a serious concern. Further studies, which take these variables into account, will need to be undertaken. Recent developments in the field of physiologically based pharmacokinetic (PBPK) modeling have led to a renewed interest in prediction and the potential DDIs in humans to guide clinical implication (Sager et al., 2015; Taskar et al., 2020). In our subsequent study, the PBPK model will be established in basic physicochemical data to predict the pharmacokinetic behavior of SOR when combined with BG in HCC patients.

In conclusion, oral concomitant administration of SOR with BG (160 mg/kg) for single and multiple doses significantly enhanced the oral bioavailability of SOR in rats. The intestinal absorption of SOR was significantly increased for BG treatment in rats in the *in situ* single-pass intestinal perfusion model. A greater understanding of potential DDIs between BG and SOR in rats may provide guidance for dosage adjustment and rational multidrug therapy for clinical applications. Nonetheless, the DDIs in rats need to be further confirmed by clinical trials in humans and patients, including clinical assessment of response to treatment.

## Data Availability

The original contributions presented in the study are included in the article/supplementary material; further inquiries can be directed to the corresponding author.

## Abbreviations

ANOVA, analysis of variance; AUC, the area under the plasma concentration–time curve; BG, baicalin; CE, collision energy; CL_z_, clearance; C_max_, the peak plasma concentration; CXP, collision cell exit potential; CYP 3A4, cytochrome P450 3A4; DDIs, drug–drug interactions; DP, declustering potential; EP, entrance potential; ESI, electrospray ionization; FDA, U.S. Food and Drug Administration; HCC, hepatocellular carcinoma; IS, internal standard; K_app_, apparent absorption rate constant; LC-MS/MS, liquid chromatography coupled to mass spectrometry; LLOQ, lower limit of quantification; MRM, multiple reaction monitoring; P_eff_, effective permeability coefficient; P-gp, p-glycoprotein; PK, pharmacokinetics; RSD, relative standard deviation; SOR, sorafenib; t_1/2_, elimination half-life; UGT1A9, uridine diphosphate glucuronosyltransferase 1A9; V_z_, volume of distribution.
